# Lung Cancer Survival in Patients With Autoimmune Disease

**DOI:** 10.1001/jamanetworkopen.2020.29917

**Published:** 2020-12-14

**Authors:** Saya Jacob, Kian Rahbari, Kyle Tegtmeyer, Jeffrey Zhao, Steven Tran, Irene Helenowski, Hui Zhang, Theresa Walunas, John Varga, Jane Dematte, Victoria Villaflor

**Affiliations:** 1 Department of Medicine, Northwestern University Feinberg School of Medicine, Chicago, Illinois; 2 Northwestern University Feinberg School of Medicine, Chicago, Illinois; 3 Northwestern University, Chicago, Illinois; 4 Division of General Internal Medicine and Geriatrics, Department of Medicine, Northwestern University Feinberg School of Medicine, Chicago, Illinois; 5 Center for Health Information Partnerships, Northwestern University Feinberg School of Medicine, Chicago, Illinois; 6 Department of Medicine, Northwestern Scleroderma Program, Northwestern University Feinberg School of Medicine, Chicago, Illinois; 7 Division of Pulmonary and Critical Care, Department of Medicine, Northwestern University Feinberg School of Medicine, Chicago, Illinois; 8 Robert H. Lurie Comprehensive Cancer Center of Northwestern University, Chicago, Illinois; 9 City of Hope Cancer Center, Duarte, California

## Abstract

**Importance:**

Patients with autoimmune disease and lung cancer pose a multidisciplinary treatment challenge, particularly with the advent of immunotherapy. However, the association between autoimmune disease and lung cancer survival is largely unknown.

**Objective:**

To determine the association between autoimmune disease and lung cancer survival.

**Design, Setting, and Participants:**

Retrospective cohort study between 2003 and 2019 at a single academic medical center (Northwestern University). A query of the Northwestern Medicine Enterprise Data Warehouse identified 349 patients with lung cancer and several autoimmune diseases. Types of lung cancers included small cell, adenocarcinoma, squamous cell carcinoma, non–small cell not otherwise specified, and large cell carcinoma. Autoimmune diseases included rheumatoid arthritis, systemic lupus erythematosus, systemic sclerosis, mixed connective tissue disease, myositis, and Sjögren syndrome. Inclusion criteria were biopsy-confirmed lung cancer, autoimmune diagnosis confirmed by a rheumatologist, and death or an encounter listed in the electronic medical record within 2 years of study end. A control group of patients with biopsy-proven lung cancer but without autoimmune disease was identified. Data analysis was conducted from March to July 2020.

**Exposure:**

Presence of autoimmune disease.

**Main Outcomes and Measures:**

Overall survival and progression-free survival in patients with autoimmune disease. The hypothesis was that patients with autoimmune disease would have worse progression-free survival and overall survival compared with patients in the control group.

**Results:**

Of the original 349 patients, 177 met inclusion criteria. Mean (SD) age at lung cancer diagnosis was 67.0 (10.0) years and 136 (76.8%) were women. Most common autoimmune diseases were rheumatoid arthritis (97 [54.8%]), systemic sclerosis (43 [24.3%]), and systemic lupus erythematous (15 [8.5%]). Most common lung cancers were adenocarcinoma (99 [55.9%]), squamous cell carcinoma (29 [16.4%]), and small cell lung cancer (17 [9.6%]). A total of 219 patients (mean [SD] age at diagnosis, 65.9 [4.1] years; 173 [79.0%]) were identified as having lung cancer without autoimmune disease and included in the control cohort. Compared with patients in the control group, patients with autoimmune disease experienced no difference in overall survival (log-rank *P* = .69). A total of 126 patients (69.5%) with autoimmune disease received standard of care vs 213 patients (97.3%) in the control group (*P* < .001). No individual autoimmune disease was associated with worse prognosis, even among patients with underlying interstitial lung disease.

**Conclusions and Relevance:**

Compared with institutional controls, patients with autoimmune disease experienced no difference in survival despite the fact that fewer patients in this group received standard-of-care treatment. No individual autoimmune disease was associated with worse prognosis. Future multicenter prospective trials are needed to further evaluate autoimmune disease and lung cancer survival.

## Key Points

**Question:**

Is there an association between autoimmune disease and lung cancer survival?

**Findings:**

This cohort study of 349 patients compared lung cancer survival in those with autoimmune disease with that of patients without it and observed no difference in overall survival, even when broken down by locoregional and distant stage. No one subtype of autoimmune disease was associated with worse survival compared with that of the control cohort, including survival of patients with underlying interstitial lung disease.

**Meaning:**

In this study, no significant difference in lung survival for patients with autoimmune disease compared with controls was observed, despite the fact that fewer patients in this group received standard-of-care lung cancer treatment.

## Teaser

This cohort study seeks to determine the association between autoimmune disease and lung cancer survival.

## Introduction

The World Health Organization reports lung cancer as globally responsible for approximately 1.76 million cancer deaths, or 18.4% of all cancer deaths.^1^ Many risk factors exist for lung cancer, including tobacco exposure, prior radiation, environmental exposures, and personal or family history of lung cancer.^2^ In addition, personal history of autoimmune disease has been implicated in the development of lung cancer.^3^ The association between autoimmune disease and cancer is thought to be bidirectional, and at times it is unclear whether autoimmune disease is a paraneoplastic phenomenon or whether the inflammatory profile and therapeutic immunosuppression in autoimmune disease lead to cancer.^3,4^

Lung cancer has been associated with several autoimmune diseases, particularly systemic sclerosis and myositis, but also rheumatoid arthritis, systemic lupus erythematosus, and mixed connective tissue disease.^3,4,5,6,7,8,9^ Systemic sclerosis is thought to portend a 3- to 4-fold greater risk of developing lung cancer, particularly in men.^10,11,12^ This risk was demonstrated in the largest North American cohort of systemic sclerosis patients.^13^ One-third to one-half of these lung cancers are adenocarcinoma.^14,15^ Patients with systemic lupus erythematosus have been shown to have a 1.28 incidence rate ratio for developing cancer, again with a male predominance.^16^ Inflammatory lung diseases, such as interstitial lung disease and idiopathic pulmonary fibrosis, have also been shown to be associated with later development of lung cancer, with some sources citing a 3- to 7-fold increased risk.^17,18,19,20^ For polymyositis and dermatomyositis, there is a 1- to 17-fold increased risk of developing cancer, including lung cancer, compared with that in the general population.^8,9,21^

The advent of immunotherapy has brought the interplay between autoimmune disease and cancer to the forefront of clinical investigation; however, the relationship between autoimmune disease and cancer survival remains ambiguous.^22^ On one hand, increased inflammation has been associated with cancer development.^23^ In rheumatoid arthritis, immune dysregulation leads to increases in many of the same cytokines and immune cells implicated in carcinogenesis.^24^ However, a previous investigation has shown increased lymphocytic activity within tumor cells, leading to improved prognosis.^25^ In small cell lung cancer, paraneoplastic autoimmune disease with anti-Hu antibodies was associated with improved survival.^26^ In patients with non–small cell lung cancer who underwent treatment with immune checkpoint inhibitors, development of treatment-related autoimmune disease was associated with increased survival.^27,28^ For this reason, there is some speculation that underlying autoimmunity may play a protective role in cancer progression.

There is mixed evidence regarding lung cancer progression in patients with autoimmune disease. Some studies suggest that cancer is overall more aggressive in this group.^29,30^ However, other studies did not observe an association between autoimmune disease and increased lung cancer mortality.^31,32^ Why autoimmune disease could portend worse lung cancer prognosis is not clear. Some studies point to long-term use of immunosuppression; however, there is conflicting evidence regarding the role of immunosuppressive treatment and risk for developing cancer.^33,34,35^

Patients with autoimmune disease and cancer pose an interdisciplinary treatment challenge and further investigation regarding natural history and treatment of disease is needed. In this study, we evaluated lung cancer survival and recurrence in patients with underlying autoimmune disease. We also characterized survival within several individual types of autoimmune disease compared with that of a control cohort. Finally, we investigated the number of patients who received standard-of-care lung cancer treatment.

The primary hypothesis of this study was that patients with autoimmune disease would have worse lung cancer overall survival and progression-free survival compared with patients with lung cancer without autoimmune disease. Secondary hypotheses in this study were that patients with autoimmune disease would have higher rates of lung cancer recurrence and that particular types of autoimmune disease would be associated with worse lung cancer survival compared with other types of autoimmune disease.

## Methods

We identified a retrospective cohort of patients within a single academic medical center, Northwestern Medicine, between 2003 and 2019. The study was performed under an approved institutional review board protocol from Northwestern University. Individual informed consent was not required given the retrospective, deidentified nature of the data collected. A query of the Northwestern Medicine Enterprise Data Warehouse was performed to identify patients with lung cancer and autoimmune disease. Lung cancer and autoimmune diseases (rheumatoid arthritis, systemic lupus erythematosus, systemic sclerosis, myositis, Sjögren syndrome, and mixed connective tissue disease) were identified with *International Classification of Diseases, Ninth Revision* (*ICD-9*) and *ICD-10 *codes and are described in the eTable in the Supplement. These particular autoimmune diseases were selected because of previously described associations with lung cancer. To be included, patients were required to have 2 *ICD* codes for autoimmune disease entered during 2 separate encounters. The *ICD* codes for autoimmune disease could be entered at any time in relation to lung cancer diagnosis, and no specific temporal relationship was required for study selection. Our initial Northwestern Medicine Enterprise Data Warehouse query identified 349 unique patients. Only patients with biopsy-proven lung cancer, autoimmune disease diagnosed by a board-certified rheumatologist, and death or an encounter documented in the electronic medical record within 2 years of the study end were included. After review, 177 patients met inclusion criteria for final analyses. Data were abstracted from the medical record by 5 reviewers (S.J., K.R., K.T., J.Z., and S.T.) consisting of 4 medical students and 1 resident physician. Abstractors were not masked to the study hypothesis. All medical record reviewers gathered standardized variables for each patient, including age at lung cancer diagnosis, sex, smoking status, date of lung cancer diagnosis, cancer histopathology findings, stage at first treatment, date of first progression from date of diagnosis as determined by imaging and clinical assessment, date of death, rates of recurrence, and types of treatments used. Stage at treatment was categorized as localized disease if there was no nodal involvement, regional disease if there was nodal involvement, and distant disease if there was evidence of metastatic sites. Presence or absence of concomitant autoimmune disease–associated inflammatory lung disease (autoimmune disease/interstitial lung disease) was determined by chest computed tomographic reports. Use of immunotherapy was noted along with general types of cancer treatment. Patients were characterized as receiving standard-of-care treatment according to stage and preset criteria (eFigure 1 in the Supplement). For patients not receiving standard of care, reasons for lack of treatment were determined as owing to either poor performance status or the existence of comorbid conditions that prevented optimal cancer treatment.

A retrospective control cohort was identified by a query of the Northwestern Medicine Enterprise Data Warehouse between 2003 and 2019. Only patients with biopsy-proven lung cancer, no prior autoimmune disease diagnosis, and death or follow-up within 2 years of the study end were included. Data were abstracted as they were for the autoimmune cohort by the same 5 abstractors.

### Statistical Analysis

Continuous variables were summarized by means and SDs and medians and interquartile ranges. Categorical variables were summarized as frequencies and percentages. Overall and progression-free survival rates were estimated via the Kaplan-Meier method and differences between autoimmune disease groups or stages were assessed via the log-rank test. Bonferroni correction was used in this instance to adjust for the numerous pairwise comparisons between the groups involved. Differences in overall and progression-free survival rates between the autoimmune disease cohort and the control cohort were assessed via the log-rank test. All tests were 2-sided, and results below α = .05 were considered statistically significant. To adjust for multiple factors in survival analyses, multivariate Cox regression was conducted under the assumption of proportional hazards. Results from these regression models were summarized as hazard ratios, their 95% CIs, and *P* values, in which hazard ratios greater than 1 indicate increased risk; those less than 1, decreased risk. Analyses were conducted in SAS version 9.4 (SAS Institute) and R version 3.6.1 (R Project for Statistical Computing).

## Results

### Autoimmune Cohort Characteristics

Of the 177 patients who were included in this study, the mean (SD) age at lung cancer diagnosis was 67 (10) years (Table 1). Most patients were women (136 [76.8%]). Most patients in the cohort had at least some smoking history (140 [79.1%]). The most common autoimmune diseases were rheumatoid arthritis (97 [54.8%]), systemic sclerosis (43 [24.3%]), and systemic lupus erythematosus (15 [8.5%]). There were 54 cases (30.5%) of autoimmune disease/interstitial lung disease. Lung cancers identified were adenocarcinoma (99 [55.9%]), squamous cell carcinoma (29 [16.4%]), small cell lung cancer (17 [9.6%]), non–small cell lung cancer not otherwise specified (13 [7.3%]), and large cell lung cancer (3 [1.7%]). There were 111 patients (62.7%) with locoregional disease at diagnosis and 58 (32.8%) with distant disease. Of the entire cohort, 139 patients (78.5%) received some type of immunosuppression for autoimmune disease before cancer diagnosis (Table 1). Of the 88 patients who underwent testing for targetable molecular alterations, a total of 40 (45.5%) were positive for *EGFR, KRAS, ALK* rearrangement, *ROS-1,* or *BRAF*. Finally, at 5 years 14 patients were lost to follow-up. Complete 5-year survival data could not be calculated for 24 patients because there was not enough time for follow-up.

**Table 1.** Patient Characteristics

Characteristic	No. (%)	
	Autoimmune disease cohort (n = 177)	Control cohort (n = 219)
Basic characteristics		
Age at lung cancer diagnosis, mean (SD), y	67.0 (10.0)	65.9 (4.1)
Women	136 (76.8)	173 (79.0)
Smoking history	140 (79.1)	172 (78.5)
Race		
White	132 (74.6)	166 (75.8)
Black	20 (11.3)	22 (10.0)
Other^a^	25 (14.1)	31 (14.2)
Lung cancer histopathology		
Adenocarcinoma	99 (55.9)	158 (72.1)
Squamous cell	29 (16.4)	30 (13.7)
Large cell	3 (1.7)	1 (0.5)
NSCLC NOS	13 (7.6)	10 (4.6)
Small cell cancer	17 (9.6)	13 (5.9)
Other	16 (9.0)	7 (3.2)
Stage at diagnosis		
Locoregional	111 (62.7)	152 (69.4)
Distant	58 (32.8)	64 (29.2)
Unstaged	8 (4.3)	3 (1.4)
Autoimmune diseases		
Systemic sclerosis	43 (24.3)	NA
Rheumatoid arthritis	97 (54.8)	NA
Autoimmune disease–associated interstitial lung disease	54 (30.5)	NA
Systemic lupus erythematosus	15 (8.5)	NA
Mixed connective tissue/overlap syndrome	12 (6.4)	NA
Myositis	11 (6.2)	NA
Sjögren syndrome	11 (6.2)	NA
Autoimmune disease treatment		NA
Prior immunosuppression	138 (78.5)	NA
No immunosuppression	38 (21.5)	NA
Standard-of-care treatment for lung cancer		
Received	126 (69.5)	213 (97.3)
Did not receive	38 (22.2)	5 (2.3)
Owing to frailty	26 (72.2)	NA
Inability to tolerate treatment	2 (5.6)	NA
Other	10 (22.2)	NA

Abbreviations: NA, not applicable; NSCLC NOS, non–small cell lung cancer not otherwise specified.

^a^ Other included individuals identifying as Asian or Hispanic or who did not specify race.

### Control Cohort Characteristics

A total of 219 patients were identified as having lung cancer without autoimmune disease and included in the control cohort (Table 1). Mean (SD) age of diagnosis was 65.9 (4.1) years, and 173 (79.0%) were women. Most patients had smoking history (172 [78.5%]). Most commonly identified lung cancers were adenocarcinoma (158 [72.1%]), squamous cell carcinoma (30 [13.7%]), small cell carcinoma (13 [5.9%]), and non–small cell carcinoma not otherwise specified (10 [4.6%]). Most patients received their diagnosis at the locoregional stage (152 [69.4%]), with a minority at distant stage (64 [29.2%]). Overall, 213 patients (97.3%) received standard-of-care treatment (eFigure 1 in the Supplement). Of the 152 patients tested for targetable molecular alterations, 89 (58.6%) were positive for *EGFR, KRAS, ALK* rearrangement, *ROS-1,* or *BRAF*. Finally, at 5 years, 19 patients were lost to follow-up.

### Standard-of-Care Treatment

According to our definition of standard of care (eFigure 1 in the Supplement), 126 patients (69.5%) in the autoimmune cohort received standard-of-care initial treatment, whereas 38 (22.2%) did not. There were 13 patients (7.3%) who did not have treatment information documented. Of the 38 patients who did not receive standard of care, 26 (68.4%) did not receive it because of poor initial performance status or frailty and 2 (5.3%) because of comorbidities of autoimmune disease. An example of this includes inability to receive radiation because of underlying pulmonary fibrosis. The remaining 10 patients (26.3%) did not undergo cancer treatment (ie, they transferred to hospice care). Of the 38 patients, 14 (36.8%) were classified as having locoregional stage cancer, 16 (42.1%) as distant stage, and 8 (21.2%) as unknown stage. In the autoimmune cohort overall, only 8 patients (4.5%) underwent treatment with immunotherapy, and in the control cohort, 5 patients (2.3%) did not receive standard-of-care therapy, all because of poor baseline functional status. Two of these patients (40.0%) had locoregional stage cancer and 2 (40.0%), distant stage. A total of 74 patients (33.8%) underwent immunotherapy. The difference in patients undergoing standard-of-care treatment between groups was statistically significant (129 [69.5%] vs 213 [97.3%]; *P* < .001). This was true in locoregional (91 [82.0%] vs 149 [98.0%]; *P* < .001) and distant (40 [67.0%] vs 62 [97.0%]; *P* < .001) stages.

### Median Survival by Stage

Table 2 shows median progression-free survival and overall survival by stage. In the autoimmune group, median progression-free survival for locoregional disease was estimated as 42.81 (95% CI, 33.18-55.62) months compared with that for the control group, which was 34.00 (95% CI, 28.26-45.47) months. For distant disease, median progression-free survival was estimated at 6.97 (95% CI, 5.16-8.44) months in the autoimmune cohort compared with 8.77 (95% CI, 6.11-12.55) months in the control cohort. Median overall survival for locoregional stage was 48.43 (95% CI, 44.55-57.00) months in the autoimmune cohort vs 39.62 (95% CI, 33.94-54.08) months in the control cohort. Median overall survival for distant stage was 9.96 (95% CI, 7.66-18.76) months and 19.09 (95% CI, 11.33-25.55) months for autoimmune and control cohorts, respectively.

**Table 2.** Survival Characteristics

Outcome^a^	Survival (95% CI), mo	
	Autoimmune disease cohort	Control cohort
Median progression-free survival		
Locoregional	42.81 (33.18-55.62)	34.00 (28.26-45.47)
Distant	6.97 (5.16-8.44)	8.77 (6.11-12.55)
Median overall survival		
Locoregional	48.43 (44.55-57.00)	39.62 (33.94-54.08)
Distant	9.96 (7.66-18.76)	19.09 (11.33-25.55)
Overall survival at 1 y , %		
Locoregional	85.41 (79.05-92.28)	87.59 (82.38-93.12)
Distant	44.05 (32.66-59.41)	58.44 (47.41-72.05)
Overall survival at 5 y, %		
Locoregional	38.14 (29.99-48.50)	36.55 (29.50-45.29)
Distant	8.51 (3.36-21.53)	12.78 (6.62-24.68)
Recurrence, No. (%)	23 (21.50)	34 (22.4)

^a^ Progression-free survival and overall survival were estimated with the Kaplan-Meier method.

### Progression-Free Survival by Stage

Progression-free survival was calculated by stage and for the autoimmune disease and control cohort (eFigure 2 in the Supplement). We observed no difference in progression-free survival between cohorts (log-rank *P* = .53). Analyses of progression-free survival by locoregional and distant stage also showed no difference in survival (log-rank *P* = .82 and log-rank *P* = .44, respectively). When analyzed with a multivariate Cox regression, only age appeared to be independently associated with worse progression-free survival (hazard ratio, 1.017; 95% CI, 1.003-1.03; *P* = .02), whereas sex, race, and smoking status were not. After adjustment with multivariate Cox regression, progression-free survival in the autoimmune cohort was not associated with any difference in survival compared with that of controls.

### Overall Survival by Stage

**Figure 1.**
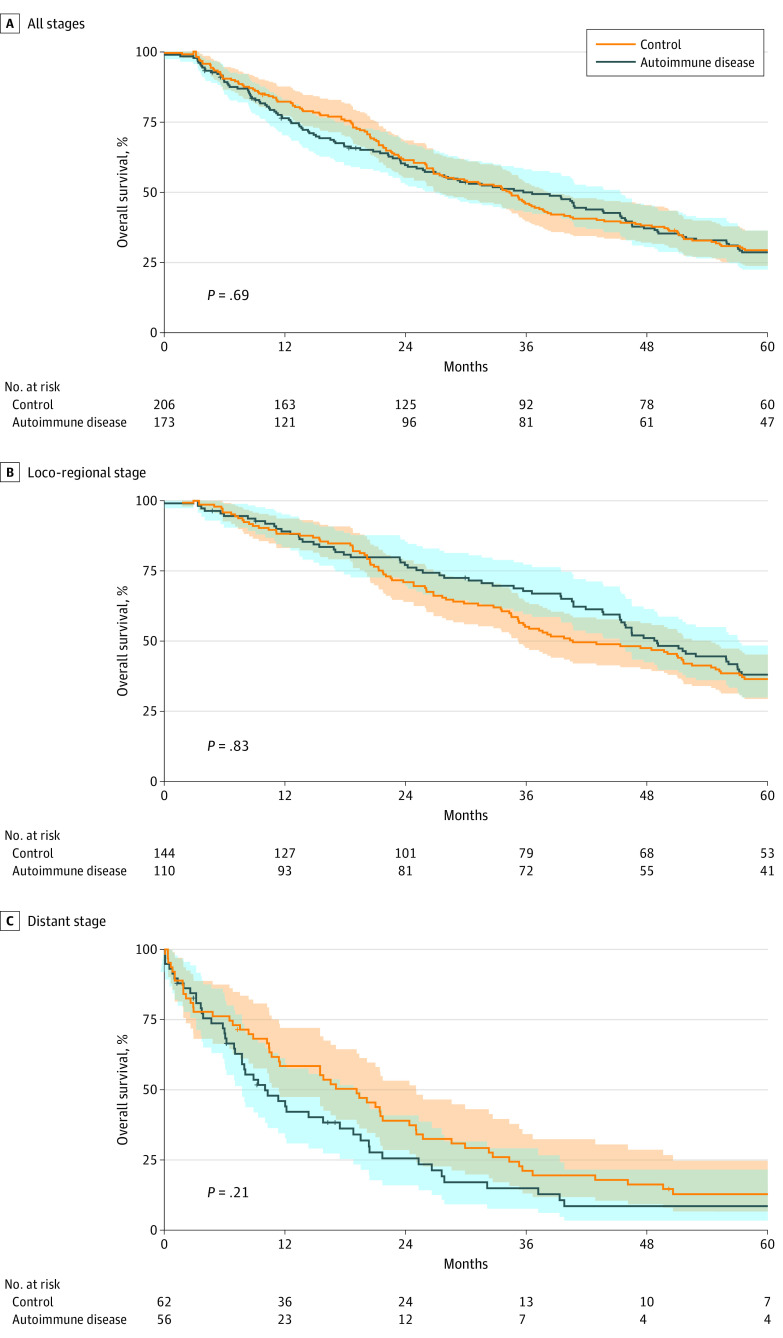
Overall Lung Cancer Survival Overall survival for autoimmune cohort was compared with that of the control group. Subgroups by stage were also compared for locoregional and distant disease. Shaded areas represent 95% CIs for each cohort.

Figure 1 shows overall survival of patients with autoimmune disease compared with that of controls. This is analyzed regardless of stage (Figure 1A), for locoregional disease (Figure 1B), and for distant disease (Figure 1C). When data were analyzed regardless of stage, patients with autoimmune disease showed no difference in survival compared with controls (log-rank *P* = .69). In locoregional disease and distant disease, we again observed no difference in survival between groups (log-rank *P* = .83 and log-rank *P* = .21, respectively). When analyzed with a multivariate Cox regression, only age appeared to be an independent risk factor for worse overall survival (hazard ratio, 1.013; 95% CI, 1.00-1.027; *P* = .049), whereas sex, race, and smoking status were not. After adjustment with multivariate Cox regression, overall survival in the autoimmune cohort was not associated with any difference in survival compared with that in controls.

### Recurrence in Locoregional Disease

For patients with locoregional disease at treatment, 23 (21.5%) had recurrence (Table 2). Overall metastatic recurrence in locoregional disease was 9.35% (10 patients). In the control cohort, there were 34 cases of recurrence (22.4%) in patients with locoregional disease.

### Survival Between Groups of Autoimmune Disease

**Figure 2.**
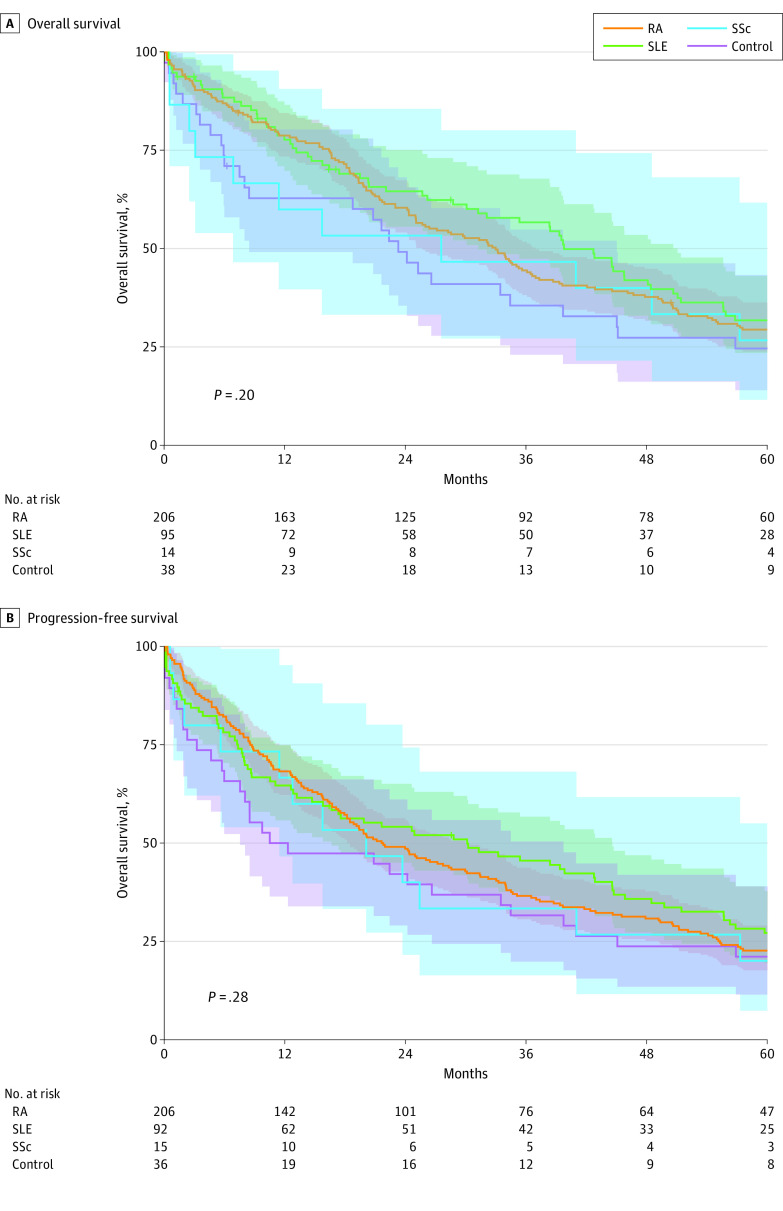
Overall and Progression-Free Survival for 3 Most Common Autoimmune Diseases Compared With That of the Control Cohort Depicted are the 3 most common autoimmune diseases (rheumatoid arthritis [RA], systemic lupus erythematosus [SLE], and systemic sclerosis [SSc]) within our cohort. Survival was calculated with the Kaplan-Meier method and showed no significant difference in overall survival or progression-free survival compared with that of the control group as assessed by the log-rank test. Shaded areas represent 95% CIs for each cohort.

**Figure 3.**
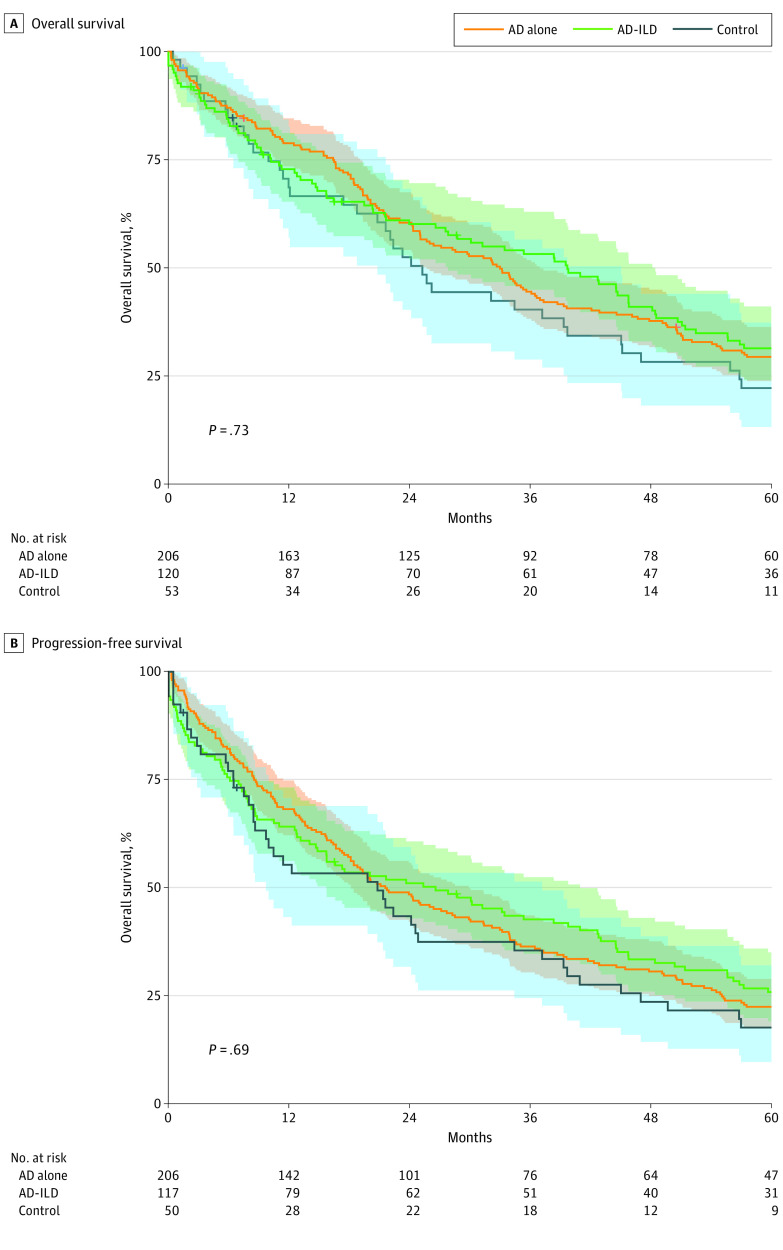
Overall and Progression-Free Survival of Patients With Autoimmune Disease/Interstitial Lung Disease vs Without It Progression-free survival and overall survival for patients with evidence of autoimmune disease–associated interstitial lung disease (AD-ILD) on computed tomographic scan. This subgroup was compared with patients with autoimmune disease without evidence of interstitial lung disease (AD alone) as well as patients in the control cohort. Comparison of survival using the Kaplan-Meier method showed no significant difference in overall survival or progression-free survival as assessed by the log-rank test. Shaded areas represent 95% CIs for each cohort.

We also performed secondary subgroup analyses of survival within individual types of autoimmune disease. No group of autoimmune disease showed statistically better or worse survival than the overall autoimmune cohort or the control cohort. Figure 2 shows progression-free survival and overall survival of the 3 most common autoimmune diseases (rheumatoid arthritis, systemic lupus erythematosus, and systemic sclerosis) compared with that of the control cohort. No significant difference in survival was observed. We also compared survival between patients with evidence of autoimmune disease/interstitial lung disease on computed tomography compared with those without it (Figure 3) and did not detect a significant survival difference in progression-free survival or overall survival. We observed no difference in overall survival for any of these subgroups at all stages, locoregional stage, or distant stage. Given the multiple subgroup analysis, Bonferroni adjustment for multiple hypothesis testing was conducted; however, we observed no difference in results after adjustment.

## Discussion

Our study observed no difference in progression-free survival or overall survival for patients with autoimmune disease and lung cancer compared with controls with lung cancer, even when adjusted through multivariate Cox regression. Similarly, both the autoimmune and control cohorts had comparable rates of recurrence in locoregional disease. Finally, subgroup analyses of individual types of autoimmune disease showed no significant difference in patients’ overall survival or progression-free survival compared with that of the control cohort, even for patients with underlying interstitial lung disease.

Despite no differences in survival, we noted a difference in the number of patients undergoing standard-of-care lung cancer treatment. Approximately 22.2% of patients in the autoimmune disease cohort did not receive standard of care compared with 5.2% of patients in the control group (*P* < .001). The most frequently observed reason for not receiving standard of care was poor underlying functional status or frailty. In the autoimmune cohort, the distribution of patients not receiving standard of care was approximately even between stages (14 patients for locoregional and 16 for distant). Furthermore, only 8 patients in the autoimmune cohort received immunotherapy compared with 74 in the control group.

The lack of difference in overall survival despite significant differences in treatment patterns between groups is intriguing and raises the possibility of a protective role of autoimmune disease. However, such a conclusion is beyond the scope of the current study. Further research is needed to fully characterize the association of autoimmune disease with lung cancer survival, particularly studies that can control for differences in treatment practices between groups. Overall, our study highlights the need for a better understanding of underlying autoimmune physiology, particularly as it may relate to survival. If a protective role of autoimmune disease on survival is indeed observed, a possible explanation is that baseline increased immunoactivity may also lead to increased immunosurveillance of developing neoplastic cells and decrease peripheral tolerance, thus impeding disease progression.^36^ Many types of autoimmune disease, including systemic sclerosis, systemic lupus erythematosus, rheumatoid arthritis, and Sjögren syndrome, have been shown to have decreased programmed cell death 1 (PD-1) activity.^37,38^ Inhibition of PD-1 is a major mechanism of immunotherapy and decreased levels lead to increased activation of T cells against host antigens.^39,40,41,42,43^ The mechanism of PD-1 downregulation in autoimmune disease is thought to be mainly through polymorphisms in the *PDCD1* gene (OMIM 600244), which is important for downstream proteins in the PD-1 pathway.^44,45^ Additionally, some patients with rheumatoid arthritis have developed antibodies against PD-1 itself.^46^ Another possible explanation may involve decreased activity of regulatory T cells.^47^ In animal models, depletion of regulatory T cells is associated with a higher incidence of autoimmune disease and has also been implicated in improved antineoplastic immune response.^48,49,50,51^ Polymorphisms in cytotoxic T-lymphocyte antigen 4 have been shown to lead to decreased regulatory T cell function and increased T cell invasion of peripheral tissue.^52,53,54^ This is of particular importance because cytotoxic T-lymphocyte antigen 4 is a target for checkpoint inhibitors.^54^ There is also evidence to support the role of the cytokine milieu of the tumor microenvironment in host recognition and response to tumors.^24,55,56,57^ An example is the cytokine interferon γ, which is known to play a role in systemic autoimmunity, particularly in the development and severity of systemic lupus erythematosus.^58^ Prior studies have demonstrated that interferon γ has led to decreased tumor development through activation of the adaptive immune response.^56,58^ It has also been implicated in improved response to treatment with checkpoint inhibitors.^56,58,59^ Thus, underlying autoimmune disease may alter T cell response and cytokine milieu, thereby allowing greater immunomediated destruction of growing tumor cells.

Recurrence rates in early-stage non–small cell lung cancer after definitive therapy have been estimated to be between 30% and 55%.^60,61,62^ In accordance with these estimates, the overall recurrence rate of 21.5% in our autoimmune cohort and 22.4% in our control cohort were slightly lower than expected. When adjusted to remove cases of small cell lung cancer, our observed recurrence rate decreased to 16.82% (18 patients).

Our analysis showed that no individual type of autoimmune disease was associated with worse survival compared with that of the group overall. We also compared survival between patients with autoimmune disease/interstitial lung disease and observed no significant survival difference between those with such disease and those without. Previous studies have shown increased cancer-related mortality in patients with interstitial lung disease compared with those without it.^63,64^ These previous studies focused on fibrotic lung disease rather than inflammatory lung disease, which may account for the difference we observed. In addition, our analysis may also have captured heterogeneity in autoimmune disease/interstitial lung disease because of varying pathophysiology and types of underlying autoimmune disease. Further studies with larger sample sizes are needed to fully characterize the association between autoimmune disease/interstitial lung disease and lung cancer mortality. To the best of our knowledge, this is the only study to compare lung cancer prognosis across multiple types of autoimmune disease.

### Limitations

There are several limitations to this study. First, it was a single-center retrospective study and thus cohort sizes were limited. This raises a particular difficulty when overall survival in subgroups of autoimmune disease is compared and limits conclusions about differences between autoimmune groups. Another limitation of this study is the possibility of introducing lead-time bias. It may be that patients with autoimmune disease undergo earlier and more frequent screening, such as screening for interstitial lung disease, which may lead to increased detection and earlier diagnosis of lung cancer than in the general population. This may result in the appearance of increased survival that is actually due to earlier detection. Furthermore, survival in both groups was better than expected compared with that of the general population, but particularly so in the control group. Median overall survival for advanced-stage non–small cell lung cancer has been reported as 7.7 to 9.3 months.^65^ We observed overall survival of 19.09 (95% CI, 11.33-25.55) months in our control group and 9.96 (95% CI, 7.66-18.76) months in the autoimmune group. Thus, it is possible that survival analyses were confounded by patient demographics and practice patterns of our institution.

## Conclusions

In this study, we observed no difference in lung cancer survival for patients with autoimmune disease compared with those in the control group. This finding is intriguing, given that fewer patients in the autoimmune cohort received standard-of-care lung cancer treatment. We compared 6 different subtypes of autoimmune disease and found that no subtype was associated with worse survival compared with that of the control cohort. This was true even for patients with evidence of interstitial lung disease. Future multicenter, prospective studies are needed to further validate our findings within both lung cancer and other tumor types. Larger studies are needed to further evaluate the association of autoimmune disease and lung cancer survival.

## Notes

Supplement. **eTable.** *ICD* Codes by Diagnosis **eFigure 1.** Standard-of-Care Treatment by Stage **eFigure 2.** PFS
