# Supplementary material for: Lung Cancer Survival in Patients With Autoimmune Disease
Source: JAMA Netw Open. 2020 Dec 14;3(12):e2029917. doi: 10.1001/jamanetworkopen.2020.29917 (PMC7737093; doi:10.1001/jamanetworkopen.2020.29917)
Supplement: Supplement. — eTable. ICD Codes by Diagnosis eFigure 1. Standard-of-Care Treatment by Stage eFigure 2. PFS [file jamanetwopen-e2029917-s001.pdf]

Supplemental Online Content

Jacob S, Rahbari K, Tegtmeyer K, et al. Lung cancer survival in patients with autoimmune disease. *JAMA Netw Open*. 2020;3(12):e2029917. doi:10.1001/jamanetworkopen.2020.29917

eTable. *ICD* Codes by Diagnosis
eFigure 1. Standard-of-Care Treatment by Stage
eFigure 2. PFS

This supplemental material has been provided by the authors to give readers additional information about their work.

© 2020 Jaco S et al. *JAMA Network Open.*

eTable. ***ICD*** **Codes by Diagnosis**

**Diagnosis**	**ICD 9**	**ICD 10**
**Systemic Sclerosis**	710.1, 517.2	M34, M34.8, M34.0, M34.1, M34.9, M35.8, M35.9, M34.89, M34.81
**Dermatomyositis/Polymyositis/Myositis NOS**	710.4, 710.3, 359.79, 359.71, 359.7	M33, M33.1, M33.0, M33.2, M33.9, M36.0, M33.09, M33.2, M33.19, M33.90, M33.01, M33.22, M33.10, M33.00, M33.11, M33.22, M33.91, M33.92, M33.99, M33.12, M33.29, M33.21, M33.02, M33.20, M33.21, M33.29, M33.9, M60.8, M60.81, M60.82, M60.83, M60.84, M60.85, M60.86, M60.87, G72.4, G72.49
**Rheumatoid Arthritis**	714, 714.81, 714.2, 714.9, 714.3, 714.32, 714.33, 714.8, 714.31, 714.4, 714.89, 714.9	M05, M06, M08.0, M08.2, M08.4, M08.8, M08.9, M08.3, M05.11, M05.12, M05.13, M05.14, M05.15, M05.16, M05.17, M05.6, M05.61, M05.62, M05.63, M05.64, M05.65, M05.66, M05.67, M05.7, M05.71, M05.72, M05.73, M05.74, M05.75, M05.76, M05.77, M05.8, M05.81, M05.82, M05.83, M05.84, M05.85, M05.86, M05.87, M06, M06.01, M06.02, M06.03, M06.04, M06.05, M06.07, M06.8, M06.81, M06.82, M06.83, M06.84, M06.85, M06.86, M06.87, M08.02, M08.03, M08.04, M08.05, M08.06, M08.07, M08.21, M08.22, M08.23, M08.24, M08.25, M08.26, M08.27, M08.41, M08.42, M08.43, M08.44, M08.45, M08.46, M08.47, M08.81, M08.82, M08.83, M08.84, M08.85, M08.86, M08.87, M08.91, M08.92, M08.93, M08.94, M08.95, M08.96, M08.97, M05.9, M06.9, M05.252, M05.329, M05.441, M05.462, M05.522, M05.652, M05.769, M05.812, M05.829, M05.859, M06.012, M06.052, M06.079, M08.259, M08.472, M08.951, M08.959, M08.969, M05.159, M05.249, M05.272, M05.279, M05.351, M05.472, M05.511, M05.539, M05.542, M05.571, M05.752, M06.069, M06.351, M06.821, M08.051, M08.059, M08.859, M05.131, M05.451, M05.549, M05.70, M05.712, M05.861, M05.151, M05.152, M05.359, M05.459,

© 2020 Jaco S et al. *JAMA Network Open.*

Diagnosis	ICD 9	ICD 10
		M05.531, M05.561, M05.60, M05.651, M05.721, M05.761, M05.762, M05.869, M05.871, M05.879, M06.80, M06.869, M08.09, M08.219, M08.249, M08.261, M08.271, M08.412, M08.422, M08.432, M05.142, M05.149, M05.161
**Sjogren's Syndrome**	710.2	M35.0, M35.01, M35.04, M35.09, M35.02, M35.03
**SLE**	710, 695.4	L93, M32, M32.1, L93.0, L93.2, M32.8, M32.9, M32.13, M32.10, M32.19
**Mixed Connective Tissue Disease/Overlap Syndromes**	710.8	M35.1
**ILD/IPAF/Pulmonary Fibrosis**	515, 516.3, 516.31, 516.9, 516.69	J84.9, J84.11, J84.89, J84, J84.1, J84.111, J84.112, J84.113, J84.17

© 2020 Jaco S et al. *JAMA Network Open.*

eFigure 1. Standard-of-Care Treatment by Stage

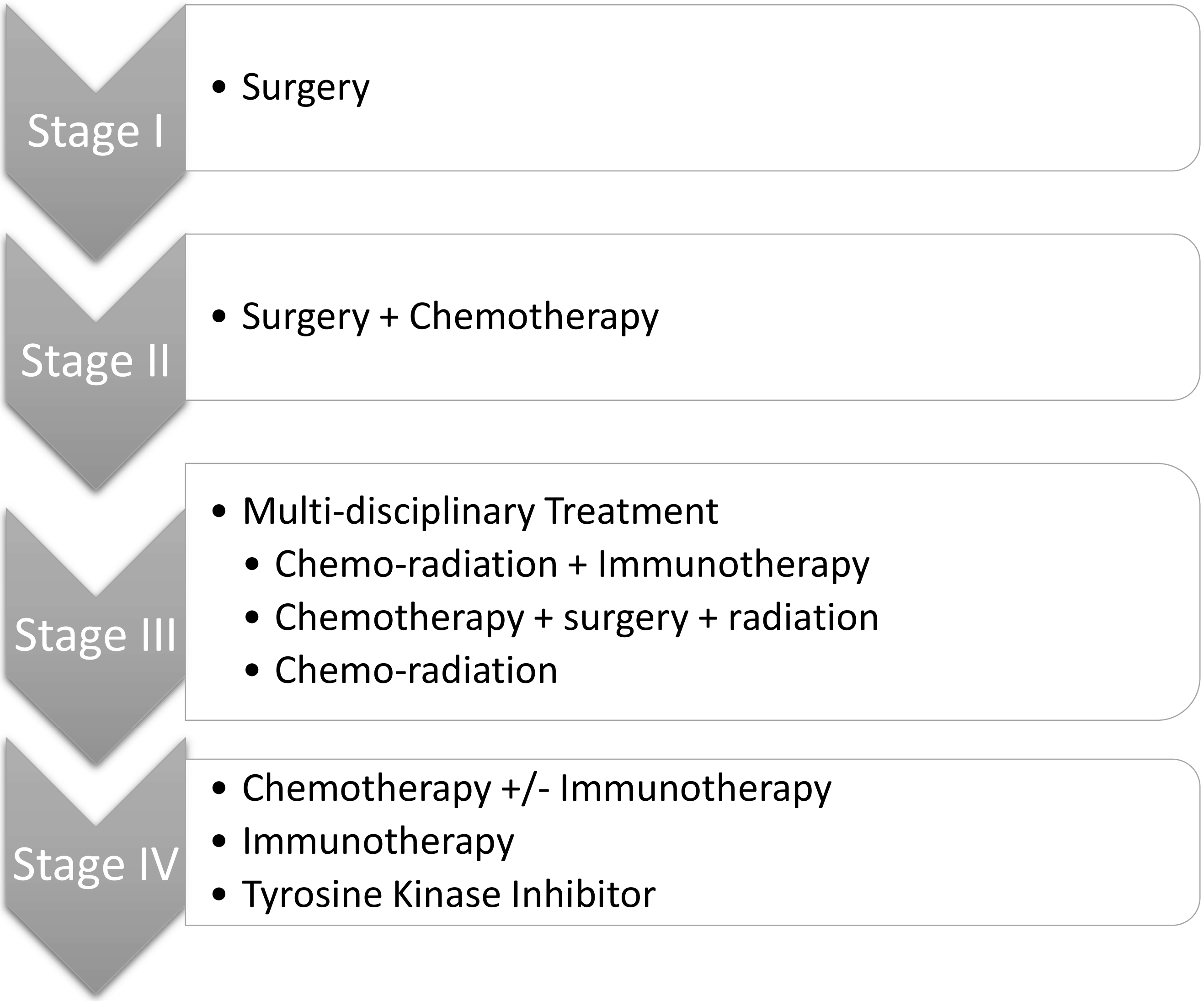

© 2020 Jaco S et al. *JAMA Network Open.*

Suppl Figure 2A: PFS, All stages

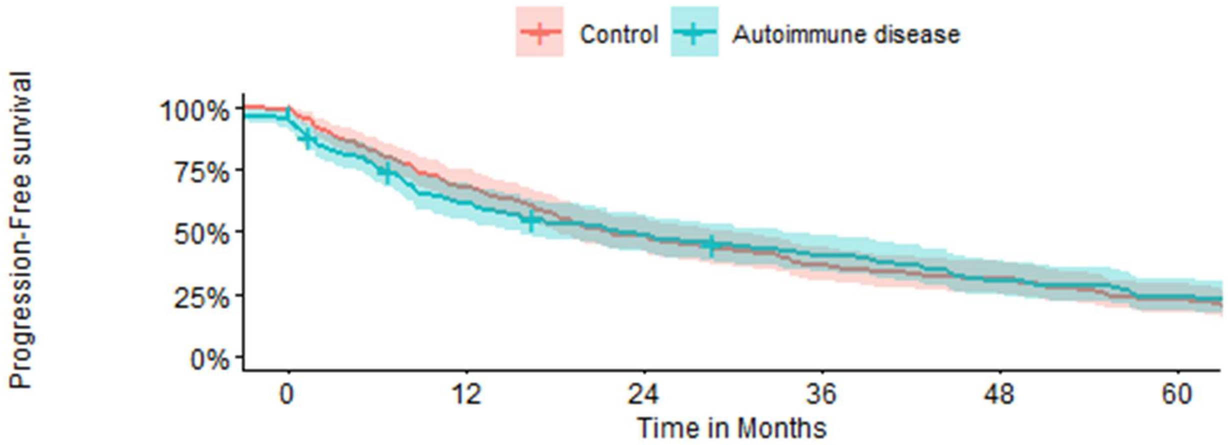

Number at risk

	0	12	24	36	48	60
Control	206	142	101	76	64	47
Autoimmune disease	167	107	84	69	52	40

Suppl Figure 2B: PFS, Loco-regional stage

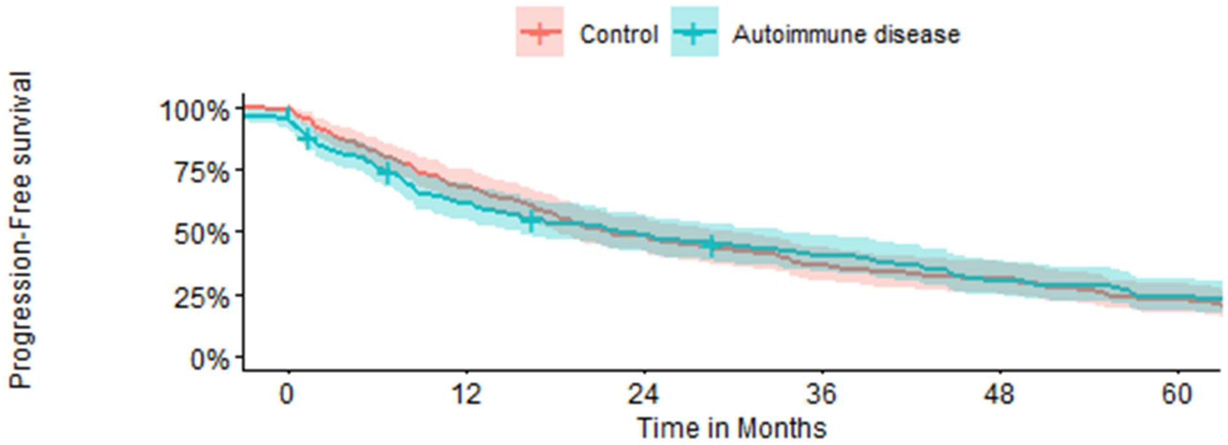

Number at risk

	0	12	24	36	48	60
Control	206	142	101	76	64	47
Autoimmune disease	167	107	84	69	52	40

© 2020 Jaco S et al. *JAMA Network Open.*

Suppl Figure 2C: PFS, Distant stage

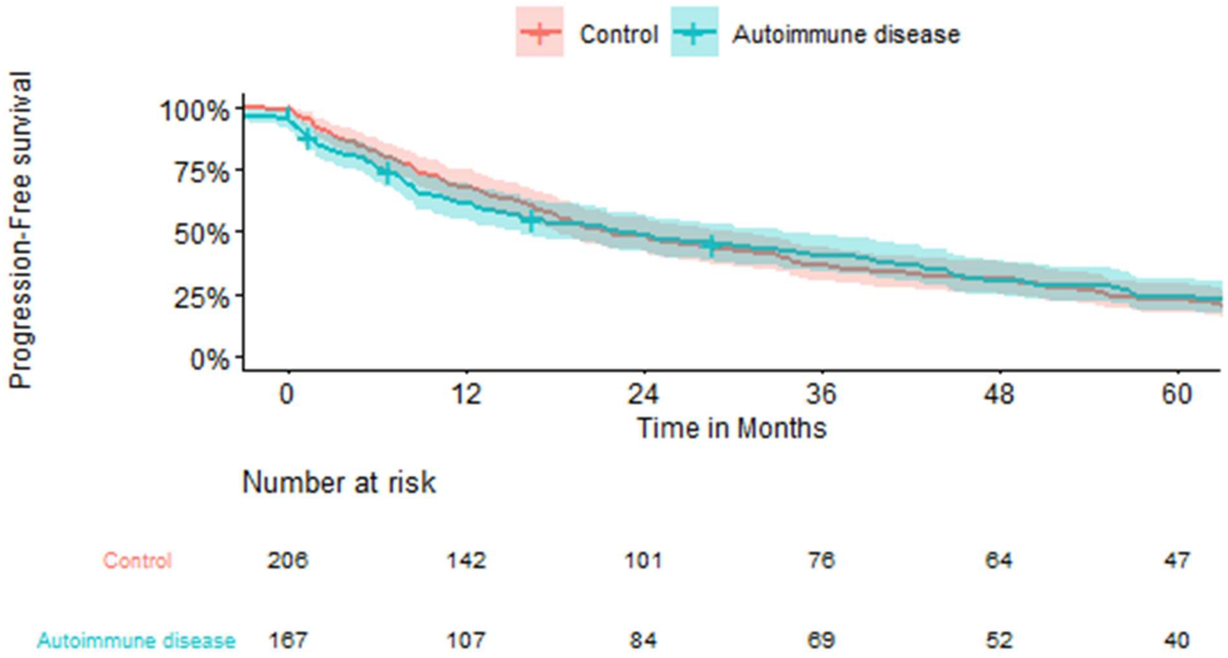

© 2020 Jaco S et al. *JAMA Network Open.*
